# Evaluated Childhood Obesity Prevention and Management Programs in Europe, 2015–2024: A Structured Narrative Review of Behavioral and Anthropometric Outcomes

**DOI:** 10.3390/nu18071100

**Published:** 2026-03-30

**Authors:** Małgorzata Wójcik, Agnieszka Kozioł-Kozakowska, Anna Iwańska, Ewelina Cichocka-Mroczek, Edyta Łuszczki, Justyna Wyszyńska, Ewa Baran, Laura González-Ramos, Isa Hartgring, Lola Martínez, Justė Parnarauskienė, Fernando Fernandez-Aranda, Augustina Jankauskienė, Dorota Drożdż, Artur Mazur, Julio Alvarez-Pitti

**Affiliations:** 1 Department of Pediatric and Adolescent Endocrinology, Chair of Pediatrics, Pediatric Institute, Jagiellonian University, 30-663 Crakow, Poland; malgorzata.wojcik@uj.edu.pl (M.W.); annaiwanska95@gmail.com (A.I.); ewa.baran@student.uj.edu.pl (E.B.); 2 Interclinical Center for the Treatment of Childhood Obesity, University Children’s Hospital, Wielicka 265 St., 30-663 Cracow, Poland; dorota.drozdz@uj.edu.pl; 3 Laboratory of Pediatric Dietetics, Department of Pediatrics, Gastroenterology and Nutrition, Jagiellonian University Medical College, 30-663 Crakow, Poland; ewelina.cichocka-mroczek@uj.edu.pl; 4 Faculty of Health Sciences and Psychology, Collegium Medicum, University of Rzeszow, 35-959 Rzeszow, Poland; eluszczki@ur.edu.pl (E.Ł.); jwyszynska@ur.edu.pl (J.W.); 5 Department of Pediatrics, Consorci Hospital General Universitari de València, 46014 Valencia, Spain; lauragonzalezramos99@gmail.com (L.G.-R.); isahartgring@gmail.com (I.H.); lolet24@hotmail.com (L.M.); japnago@gmail.com (J.A.-P.); 6 iPEDITEC, Fundación Investigación Hospital General Universitario de Valencia, 46014 Valencia, Spain; 7 CIBER Physiopathology of Obesity and Nutrition (CIBERobn), Health Institute Carlos III, 28029 Madrid, Spain; ffernandez@bellvitgehospital.cat; 8 Clinic of Children’s Diseases, Institute of Clinical Medicine, Faculty of Medicine, Vilnius University, LT-08661 Vilnius, Lithuania; juste.parnarauskiene@santa.lt (J.P.); augustina.jankauskiene@santa.lt (A.J.); 9 Clinical Psychology Department, Bellvitge University Hospital, 08907 Barcelona, Spain; 10 Psychoneurobiology of Eating and Addictive Behaviors Group, Neuroscience Program, Bellvitge Biomedical Research Institute (IDIBELL), 08908 Barcelona, Spain; 11 Department of Clinical Sciences, School of Medicine and Health Sciences, University of Barcelona, 08907 Barcelona, Spain; 12 Departament of Pediatric Nephrology and Hypertension, Chair of Pediatrics, Pediatric Institute, Jagiellonian University, 30-663 Crakow, Poland; 13 Institute of Medical Sciences, Medical College of Rzeszow University, 35-959 Rzeszow, Poland; armazur@ur.edu.pl

**Keywords:** childhood obesity, prevention, Europe, intervention programs, physical activity, diet, behavioral outcomes

## Abstract

Background: This structured narrative review summarizes and critically appraises evaluated childhood obesity prevention programs implemented in European countries and published between 2015 and 2024. Methods: Systematic searches for PubMed, EBSCOhost, and Google Scholar, complemented by research registries, were conducted year-by-year and independently screened by two reviewers. Results: Five multinational/international programs were identified alongside multiple national initiatives delivered in family, school, community, healthcare, and digital settings. Overall, interventions consistently improved intermediate outcomes—such as selected dietary behaviors, physical activity participation, knowledge, and parental self-efficacy—more than anthropometric endpoints. Effects on BMI/BMI z-score or overweight/obesity prevalence were heterogeneous and frequently small or non-significant, especially for short-duration, single-setting educational interventions. More favorable anthropometric outcomes were commonly reported in long-term, population-scaled physical activity or community-based programs as well as in multidisciplinary healthcare-supported approaches; however, these strategies were typically resource-intensive and sometimes showed differential effectiveness across socioeconomic or cultural groups. Conclusions: The evidence indicates that single-setting or short-term interventions may improve selected behavioral outcomes but are generally insufficient to produce sustained effects on anthropometric measures without integration into broader, multi-level strategies. It is needed to integrate families, schools, communities, and health services with explicit attention to sustainability and equity. Technology-supported tools may strengthen reach and continuity when embedded within comprehensive prevention frameworks.

## 1. Introduction

Childhood obesity constitutes one of the most urgent public health challenges in Europe and globally. Over the last four decades, the prevalence of overweight and obesity among children and adolescents has risen substantially [1]. Excess body weight in early life is strongly associated with increased risk of somatic and psychosocial disorders, as well as persistence of obesity into adulthood with negative health consequences [2]. These facts have led to increased awareness of the need to implement actions that could reverse the epidemiological trend. Therefore, at various organizational levels, attempts are being made to implement preventive programs for individuals at risk of developing childhood obesity. However, to date, no single effective universal model has been established. This narrative review evaluates childhood obesity prevention programs implemented in European countries between 2015 and 2024, with a focus on intervention scope, target populations, and reported outcomes.

Nutritional education programs in childhood obesity prevention typically aim to improve dietary behaviors, physical activity, and health-related knowledge, while also supporting long-term weight regulation. These interventions may include educational strategies, behavior change techniques, environmental modifications, and policy-level actions. Increasingly, multicomponent approaches integrating family, school, community, and healthcare settings are considered the most effective framework for achieving sustainable outcomes.

In this review, we use the term ‘program’ to refer to evaluated interventions implemented in European settings that aim to prevent excess weight gain and/or improve obesity-related behaviors in children and adolescents. Outcomes of interest included anthropometric endpoints (e.g., BMI/BMI z-score or overweight/obesity prevalence) and intermediate behavioral outcomes (dietary intake, physical activity, sedentary behaviors, and related knowledge/attitudes).

## 2. Methodology

This study was conducted as a structured narrative review with a transparent, pre-defined search and selection process, followed by narrative (non-meta-analytic) synthesis of findings. The review covered the period 1 January 2015 to 31 December 2024 and focused on evaluated childhood obesity prevention programs implemented in European countries.

This review was designed as a structured narrative synthesis. This approach was selected due to the substantial heterogeneity across included studies in terms of intervention types (e.g., educational, behavioral, environmental, and multicomponent), target populations (universal vs. high-risk groups), outcome measures (behavioral vs. anthropometric), and study designs (randomized, non-randomized, and observational).

Given this diversity, quantitative synthesis or strict systematic review methodologies with meta-analysis were not considered appropriate, as they could obscure important contextual differences between interventions. Instead, a structured narrative approach allowed for a transparent, systematic identification of studies combined with an interpretative synthesis of findings.

### 2.1. Information Sources and Search Strategy

We performed systematic, year-by-year searches in PubMed, EBSCOhost (databases: 2015–2024), and Google Scholar. Searches were last updated on [1 December 2025]. In addition, we searched and screened the reference lists of included papers to identify additional eligible studies. To improve transparency and reproducibility, the search strategy was predefined and applied consistently across databases. Searches combined controlled vocabulary (e.g., MeSH terms where applicable) and free-text terms related to population, condition, and intervention.

A representative search string included combinations such as (“child” OR “adolescent” OR “preschool”) AND (“obesity” OR “overweight”) AND (“prevention” OR “intervention” OR “program”) AND (“Europe” OR names of individual European countries).

Where necessary, search terms were adapted to the syntax of individual databases. The full search strategy is available upon request.

### 2.2. Eligibility Criteria

We included peer-reviewed publications that (1) reported an evaluation of a program/intervention implemented in a European setting; (2) targeted children and/or adolescents aged 0–18 years (with or without family involvement); and (3) reported at least one outcome relevant to obesity prevention, including anthropometric endpoints (e.g., BMI, BMI z-score/BMI-SDS, overweight/obesity prevalence, body composition measures) and/or intermediate behavioral outcomes (e.g., dietary behaviors, physical activity, sedentary behaviors, lifestyle-related knowledge/attitudes, parental self-efficacy). We included randomized and non-randomized controlled studies as well as observational evaluations with pre/post or trend analyses where outcome data were available. We excluded studies conducted outside Europe, protocols without results, editorials/commentaries, and publications without evaluative outcome data. Language restrictions were English only.

### 2.3. Study Selection

**Figure 1:**
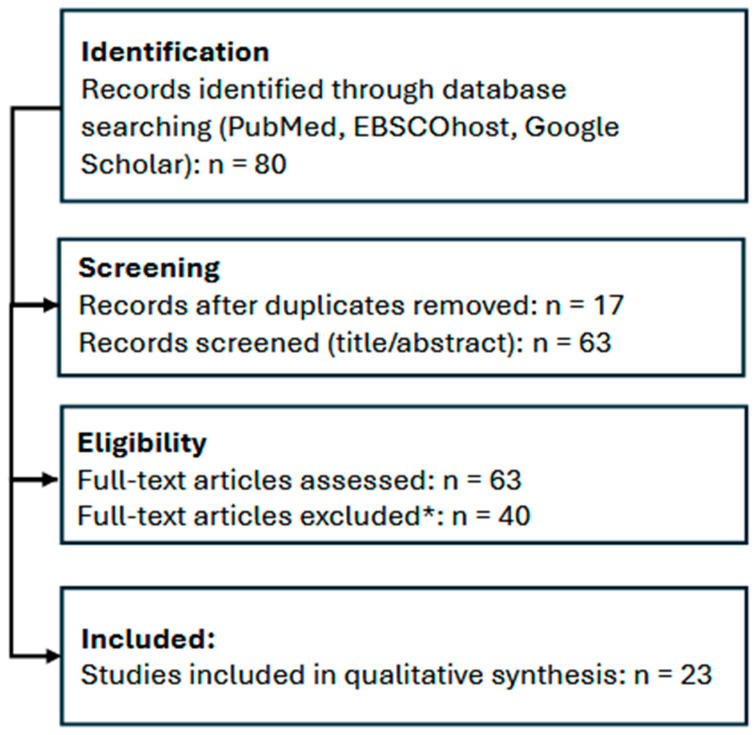
Prisma Flow Diagram. * Studies excluded due to lack of key information required for analysis or because they were conducted outside Europe.

All records were screened independently by two reviewers in two stages: (1) title/abstract screening and (2) full-text assessment. Discrepancies were resolved through discussion and consensus (Figure 1).

### 2.4. Data Extraction and Critical Appraisal

A standardized extraction framework was used to capture country, target group, setting (family/school/community/healthcare/digital), intervention components, duration and intensity, study design, sample size, follow-up, and reported outcomes. Given the heterogeneity of interventions and evaluation designs, we did not conduct a formal risk-of-bias assessment using a single tool; instead, we documented key methodological limitations reported by authors and study-design constraints (e.g., lack of control group, short follow-up, implementation fidelity issues) and considered these factors when interpreting findings.

A formal risk-of-bias assessment using a single standardized tool (e.g., Cochrane RoB) was not performed. This decision was based on the substantial heterogeneity of included studies, which encompassed randomized controlled trials, non-randomized interventions, and observational evaluations.

As no single tool is appropriate for consistently assessing bias across such diverse designs, applying one instrument could result in misleading or non-comparable assessments. Instead, key methodological limitations reported in the original studies were extracted and considered in the interpretation of findings.

### 2.5. Data Synthesis

We conducted a narrative synthesis and organized results by intervention setting/type: multinational/international programs, parent-focused, school-based, community-based, healthcare-supported, and technology-based interventions. For each category, we summarized patterns of effectiveness separately for behavioral outcomes and anthropometric outcomes, highlighting consistency/heterogeneity of findings, sustainability of effects when follow-up was available, and any evidence of differential effectiveness across socioeconomic or cultural groups. Meta-analysis was not performed due to substantial heterogeneity in interventions, outcomes, and study designs.

### 2.6. Methodological Appraisal

In addition to the narrative synthesis, a structured methodological appraisal of the included studies was conducted. Each study was descriptively assessed based on study design (e.g., randomized controlled trial, non-randomized controlled study, or observational evaluation), presence and type of control/comparator group, duration of follow-up, and sustainability of reported effects.

Methodological robustness was qualitatively classified based on predefined criteria, including study design (e.g., randomized vs. non-randomized), presence and type of control group, sample size, and duration of follow-up. Studies meeting more of these criteria (e.g., randomized design with long-term follow-up) were categorized as higher robustness. This classification was intended to support comparative interpretation rather than to replace formal risk-of-bias assessment.

The findings of this appraisal are presented in Table 1. No formal risk-of-bias assessment tool (such as Cochrane RoB) was applied, as the review included heterogeneous study designs and aimed to provide a comparative overview rather than a quantitative meta-analysis.

## 3. Results

A total of 23 evaluated programs were included in this review, comprising multinational (n = 5), parent-focused (n = 6), school-based (n = 8), community-based (n = 3), healthcare-based (n = 1), and technology-based interventions (n = 2). Most studies employed controlled designs, although a substantial proportion were non-randomized or observational. Intervention duration ranged from 3 months to several years, with only a minority including long-term follow-up (see Table 2).

### 3.1. Multinational and International Programs

Five programs with an international scope encompassing European countries, including one conducted jointly with Brazil, have been identified [5,6,7,8,9]. In two cases, the target groups were children together with their families. Two programs were specifically addressed to individuals with overweight or obesity, while the remaining had an educational and preventive character and were addressed to a broad population regardless of weight status. Finally, two of them were dedicated to preschool children under six years of age.

The Feel4Diabetes intervention demonstrated partial effectiveness, improving selected lifestyle behaviors in families at risk for type 2 diabetes, including increased breakfast and fruit consumption in certain countries and improved BMI z-scores among children with overweight. However, no significant effects were observed in children with obesity, nor were improvements detected in overall physical activity or multiple targeted lifestyle behaviors [5]. The ToyBox intervention resulted in a greater reduction in prepacked fruit juice consumption among preschool children in the intervention group compared with controls. While this represents a relevant public health outcome, a concurrent reduction in plain milk consumption was also observed [6]. The BigO study indicated that a personalized, multidisciplinary lifestyle intervention improved specific dietary behaviors in children and adolescents, including increased daily water intake between meals and reduced consumption of evening snacks while watching television [7]. The Med4Youth study reported that adolescents following a Mediterranean diet experienced greater reductions in BMI, body weight, and hip circumference, as well as improvements in blood pressure and fat distribution, compared with peers following a low-fat diet. Improvements in cardiometabolic markers were predominantly observed in the Mediterranean diet group [8]. The OCARIoT experience demonstrated particularly promising outcomes, with a reduction in obesity prevalence in 75.5% of participants relative to baseline. However, some concerns arise due to the inconsistency between the data presented in the ‘Results’ section and the conclusions drawn. The intervention integrated multimodal datasets and a decision-support system based on Internet of Things technology, engaging children, educators, and health care professionals throughout the intervention process [9].

### 3.2. Parent-Focused Interventions

The PROBIT trial evaluated an educational program delivered to parents from birth until two years of age. It was conducted in northern Italy and offered the parents of newborns an educational program. The authors found that the program did not significantly reduce overweight or obesity in children at age two, despite a non-significant trend toward lower obesity prevalence in the intervention group. However, the program did improve early feeding practices, particularly responsive, on-demand feeding at three months. The findings indicate that while parental education may influence feeding behavior, larger and more powerful studies are needed to determine its effectiveness in preventing early obesity [10]. A highly interesting set of findings was presented following the analysis of the English preventive program for preschool children: Health Exercise Nutrition for the Really Young (HENRY). Based on the evaluation, the eight-week educational program dedicated to parents (n = 1100) led to significant improvements in parenting confidence, family eating behaviors, dietary intake, and physical activity among participating families [27]. The project was grounded in a theory-based, community-delivered model that equips parents of preschool children with skills and knowledge to support healthier family lifestyles. Its core assumption is that enhancing parental self-efficacy, establishing positive routines, and promoting balanced nutrition and activity in early childhood can contribute to long-term obesity prevention [4]. In the Danish ”Boost” study targeting children at the age of 13, higher levels of parental involvement in a multi-component school-based intervention were associated with significantly greater fruit and vegetable intake among adolescents. While the dose delivered of parental components was low to moderate, most parents appreciated the intervention and engaged in discussions with their children. These findings suggest that parental involvement can enhance the effectiveness of dietary interventions, provided implementation challenges are addressed [11]. Different results were obtained in the German EPOC program (Empowering Parents of Obese Children). This intervention was addressed to parents of 686 children (7–13 years old). The project evaluated whether adding a brief, behaviorally oriented parent-training program to inpatient rehabilitation for children with obesity would improve long-term weight outcomes. Although both groups showed meaningful reductions in BMI-SDS and improvements in lifestyle and quality of life, the additional parent training did not provide benefits beyond those achieved with written information alone [12]. The Healthy School Start study, which was conducted in Stockholm County, focused on the role of parental support in promoting children’s health behavior. The trial found no sustained intervention effects on children’s diet, screen time, physical activity, or BMI-SDS four years after the program. Only limited subgroup effects emerged, and these were either not robust or not clinically meaningful. Overall, the findings indicate that the intervention did not produce lasting benefits and highlight the need for more intensive, sustained, and better-integrated school-based obesity-prevention strategies [13]. The German cluster randomized trial (primary school children aged 9 to 12 years and their parents, from 82 schools) showed that the motion-controlled “Kids Obesity Prevention” game significantly improved and sustained nutrition and stress-coping knowledge in children compared with a brochure-based control. Children demonstrated the capacity to accurately apply the principle of dietary energy density under conditions of temporal constraint, reflecting a robust understanding of fundamental nutritional concepts. However, these cognitive gains did not translate into observable behavioral changes [15]. The “PETICA-Play for Health” school-based program in Croatia significantly improved pupils’ knowledge and promoted healthier lifestyle habits, particularly in rural settings, including increased physical activity, breakfast consumption, and vegetable intake, along with reduced sweets and salty snack consumption. The study also highlighted the influence of parents as role models for children’s physical activity. These results indicate that the PETICA program is an effective educational approach supporting childhood obesity prevention [16]. A pooled analysis of three school-based family support trials in Sweden found that the program produced small overall reductions in the BMI z-score of the participants, with a clinically meaningful decrease in children with obesity at baseline (−0.21), especially among those with a non-Nordic parent (−0.24). The effects were not sustained during the 5–6 months post–intervention period, indicating that ongoing support may be needed to maintain benefits. These findings suggest that universal school-based family programs can achieve BMI changes comparable to targeted behavioral obesity treatments [28]. A Swedish cluster randomized trial (PRIMROSE) found that a long-term, motivational interviewing-based parental support program delivered through child health services did not produce significant effects on children’s weight-related outcomes at the 1-year follow-up. Although some parental characteristics appeared to moderate outcomes, these effects were small and not statistically robust. Overall, the results confirm the absence of both immediate and delayed intervention effects and highlight the need to investigate additional individual and contextual determinants of prevention success [14]. In a 6-month mHealth obesity prevention study among Swedish preschool-aged children, no significant effect was observed on fat mass index between intervention and control groups. However, the intervention group showed a significant improvement in a composite score of dietary and physical activity behaviors, particularly among children with a fat mass index above the median. These findings suggest that mobile health interventions may positively influence health behaviors, even if effects on body composition are not immediately apparent [25].

### 3.3. School-Based Interventions

The WAVES study, conducted in 200 schools in the UK, a large cluster of randomized controlled trials, evaluated a 12-month school- and family-based lifestyle program aimed at promoting healthy eating and physical activity among young children. Across more than 1400 pupils, the intervention produced no statistically significant reductions in BMI z-score at either 15 or 30 months nor improvements in secondary anthropometric, dietary, physical activity, or psychological outcomes. Effect estimates were small and nonsignificant, indicating that this experiential, school-delivered approach did not prevent excess weight gain. Overall, the findings suggest that such school-based programs alone are unlikely to substantially influence the childhood obesity epidemic [17]. The large-scale, real-world school-based physical activity intervention in Slovenia effectively reduced BMI and helped prevent and treat obesity among children aged 6–14 years. Benefits increased with longer participation, with maximal effects after 3–4 years and the greatest impact observed in children with obesity at baseline. The findings demonstrate that sustained, population-level school PA programs can meaningfully improve weight outcomes, particularly for children at the highest risk [18]. An Italian study based on five-month multidisciplinary intervention combining physical education and nutritional guidance produced modest but meaningful behavioral changes in schoolchildren. Although body fat percentage increased slightly across all groups, both experimental groups demonstrated higher weekly physical activity levels and reduced sedentary time, with children with obesity remaining the most sedentary. Dietary patterns also improved, with significant changes in the consumption of specific foods. Overall, the findings support the potential of integrated school-based programs to promote healthier habits, while highlighting the need for continued refinement to sustain long-term lifestyle improvements [3]. In Northern Ireland, a 12-week, teacher-led, classroom-based activity-break intervention produced a significant increase in accelerometer-measured weekday moderate-to-vigorous physical activity (MVPA) in primary school children (n = 120), although it did not improve BMI and was accompanied by an increase in skinfold thickness. These findings suggest that short, structured in-class activity bouts can enhance daily physical activity but may not be sufficient to influence adiposity [3]. The “Extra Fit!”, a 12-week, teacher-led, classroom-based activity break program, produced a significant increase in children’s weekday MVPA, adding approximately 9.5 min of MVPA per day compared with controls. Despite this improvement, no changes in BMI were detected, and the intervention group demonstrated an increase in skinfold thickness. These findings indicate that short, structured activity breaks can effectively elevate daily physical activity but may be insufficient on their own to influence adiposity. Overall, the program represents a promising strategy for helping children achieve recommended MVPA targets [20]. An interesting attempt was the AHEAD program (Activity and Healthy Eating in ADolescence), a peer-led, school-based intervention implemented in England (n = 928, 12–13 years old). Unfortunately, unlike similar initiatives aimed at smoking cessation, the program dedicated to promoting healthy eating and physical activity did not yield the expected results [19].

### 3.4. Community-Based Interventions

The Spanish, cross-sectional study evaluating the THAO Salud Infantil community-based program (n = 27,686, 3–12 years old) found a downward trend in childhood overweight and obesity over 2009–2019. Anthropometric improvements were particularly observed in girls, while children from lower-income families remained at higher risk and benefited less from the intervention. Overall, the findings support the effectiveness of community-based strategies in preventing childhood overweight and obesity [21]. A 7-year community-based intervention in six French cities led to a significant reduction in overweight and obesity among preschool (18.1% → 13.0%) and primary school children (20.9% → 16.9%) between 2008 and 2015. The decrease was consistent across sex and school priority education areas. These findings indicate that long-term, community-based programs can effectively reduce childhood obesity prevalence and support the promotion of healthy habits from an early age [22]. An exploratory study that examined the recruitment, assessment and intervention evaluation processes for obesity prevention among minority ethnic children in London sheds new light on the issue. The study highlights both logistical challenges and opportunities across schools and places of worship. Despite lower recruitment rates in faith-based settings, multi-strategy engagement enabled effective participation, and high completion rates were achieved for most dietary and physical activity measures. Limitations in nutrient composition data for culturally specific foods were identified, underscoring measurement challenges in diverse populations. While schools provided more consistent logistics, places of worship offered valuable access to children and families, supporting the need for community-based participatory approaches that integrate cultural contexts into intervention design [23].

### 3.5. Programs Involving Resources of the Healthcare System

The prospective study of 2400 children and adolescents in Greece demonstrated that the National e-Health Program for the Prevention and Management of Overweight and Obesity significantly reduced obesity prevalence by 32.1% and overweight prevalence by 26.7% after one year. Participants also showed marked improvements in cardiometabolic risk factors. These results indicate that a personalized, multidisciplinary, e-health–based approach can be an effective strategy for managing childhood and adolescent overweight and obesity at scale [24].

### 3.6. New Technology-Based Interventions

Mobile health interventions, including the MINISTOP and MINISTOP 2.0 trials, improved composite dietary and physical activity behavior scores, and parental self-efficacy. These Swedish real-world trials demonstrated that the mHealth app effectively improved children’s dietary and lifestyle behaviors over six months, including reduced intake of sweet and savory treats, sweet drinks, and screen time. Parents in the intervention group also reported higher self-efficacy for promoting healthy eating and physical activity. While no significant changes were observed in children’s BMI, high parental satisfaction and engagement support the feasibility and potential of implementing the app in routine child health care [26]. To facilitate comparison across intervention types, the key characteristics and outcomes of the childhood obesity prevention programs reviewed are summarized in Table 1.

## 4. Discussion

The present review highlights the substantial heterogeneity in the effectiveness of childhood obesity prevention programs implemented in Europe. While improvements in intermediate outcomes—such as dietary behaviors, physical activity, and health-related knowledge—were frequently observed, consistent and sustained effects on anthropometric outcomes remained limited. Importantly, this variability should not be interpreted solely as inconsistency of evidence but rather as a reflection of the complexity of obesity as a multifactorial condition requiring context-sensitive and sustained interventions [1,2]. At the same time, part of this heterogeneity may also arise from methodological differences across studies, including variation in study design, outcome definitions, and follow-up duration.

One of the most consistent patterns identified across studies is the relationship between intervention intensity, duration, and outcomes. Short-term and low-intensity interventions, particularly those limited to educational components, were generally associated with improvements in knowledge and selected behaviors but rarely translated into measurable changes in anthropometric indicators [3,15,16,20].

In contrast, interventions characterized by longer duration and higher intensity—especially those sustained over several years or embedded in routine systems—demonstrated more favorable anthropometric outcomes, as observed in population-level and long-term community-based programs [18,21,22]. However, interpretation of these findings should take into account that longer and more intensive programs are often evaluated using non-randomized or observational designs, which may introduce bias and limit causal inference. Moreover, differences in follow-up periods across studies make direct comparison challenging.

Another key explanatory dimension relates to the degree of integration across settings. Interventions implemented within a single setting—such as schools or family environments—were generally less effective in producing sustained anthropometric changes [17,20]. While they often improved specific behaviors, these effects were likely attenuated by external influences beyond the intervention scope.

More favorable outcomes were observed in interventions integrating multiple settings, such as school, family, community, and healthcare systems, including large-scale multinational or healthcare-supported programs [5,9,24]. These approaches appear better suited to address the complex and interacting determinants of obesity. However, such programs are typically resource-intensive, and their implementation varies substantially across contexts, which may affect both effectiveness and reproducibility. In addition, limited reporting of implementation fidelity and adherence in primary studies makes it difficult to determine which specific components drive observed effects.

The timing of intervention also emerged as an important factor influencing effectiveness. Programs targeting younger children, particularly in the preschool and early school-age period, tended to show more favorable behavioral outcomes, likely due to greater parental influence and higher behavioral plasticity [4,10,14].

Nevertheless, even in early-life interventions, sustained anthropometric effects were not consistently observed [10,13,14]. This may reflect both the need for longer follow-up and the methodological constraints of included studies, many of which were not designed to capture long-term outcomes. In older children and adolescents, additional complexity arises from developmental changes, increasing autonomy, and environmental influences, which may reduce responsiveness to single-component interventions [11,13].

A consistent finding across intervention types was the discrepancy between improvements in behavioral outcomes and limited effects on anthropometric measures [4,10,11,12,13,14,15,25,27,28]. This gap may reflect insufficient intervention duration, compensatory behaviors outside intervention settings, and variability in outcome measurement.

Importantly, commonly used anthropometric indicators such as BMI and BMI z-score have recognized limitations, particularly in children undergoing growth and pubertal development [1]. Differences in how outcomes were measured and reported across studies further complicate interpretation and comparison. These methodological issues may partially explain why behavioral improvements do not consistently translate into measurable changes in body composition.

Technology-based interventions, including mobile health and digital tools, demonstrated promising effects on behavioral outcomes, engagement, and parental involvement [7,25,26]. Their scalability and potential for personalization make them attractive components of contemporary prevention strategies.

However, their effects on anthropometric outcomes remain limited, particularly when implemented as standalone interventions [25,26]. In addition, most available studies are short-term and vary in design and evaluation methods, which limits the ability to draw firm conclusions regarding long-term effectiveness.

Several studies reported differential effectiveness across socioeconomic and cultural groups, with children from disadvantaged backgrounds often benefiting less from universal interventions [21,23]. This highlights the importance of structural determinants of health, including access to healthy foods, opportunities for physical activity, and broader socioeconomic conditions.

However, interpretation of these findings is constrained by limited stratified analyses and inconsistent reporting across studies. As a result, the extent to which interventions contribute to reducing or exacerbating health inequalities remains insufficiently understood.

## 5. Implications for Interpretation, Research, and Practice

The findings of this review should be interpreted in light of several methodological considerations. The included studies were highly heterogeneous in terms of design, intervention characteristics, and outcome measures, which precluded quantitative synthesis and limited direct comparability. In addition, the absence of a formal risk-of-bias assessment and reliance on published data may introduce uncertainty in the strength of evidence.

Despite these limitations, the structured narrative approach adopted in this review allows for the identification of consistent patterns across diverse real-world interventions. The evidence suggests that single-setting or short-term interventions may improve selected behavioral outcomes but are generally insufficient to produce sustained effects on anthropometric measures [17,20].

The evidence indicates that single-setting or short-term interventions may improve selected behavioral outcomes but are generally insufficient to produce sustained effects on anthropometric measures without integration into broader, multi-level strategies. It is needed to integrate families, schools, communities, health services, and policy frameworks with explicit attention to sustainability and equity.

More effective prevention strategies are likely to require long-term, multi-level approaches integrating family, school, community, and healthcare components [18,21,22,24]. Future research should prioritize standardized outcome measures, longer follow-up periods, and improved reporting of implementation processes to better understand which intervention components are most effective and sustainable.

It is important to acknowledge that BMI and BMI z-score, although widely used in epidemiological studies, have recognized limitations, particularly in adolescents undergoing pubertal development, where changes in body composition and growth velocity may affect interpretation. Therefore, anthropometric outcomes reported in the reviewed studies should be interpreted with caution, and future interventions should incorporate more precise measures of adiposity where feasible.

## 6. Conclusions

Effective prevention of childhood obesity in Europe requires long-term, multilevel, and integrated approaches that combine family, school, community, and technological components.

Across evaluated European interventions published between 2015 and 2024, improvements were more consistently reported for intermediate outcomes (knowledge, attitudes, and selected dietary or physical activity behaviors) than for anthropometric endpoints such as BMI/BMI z-score or overweight/obesity prevalence. Short-duration, single-setting educational programs rarely translated into durable changes in adiposity. In contrast, more promising anthropometric signals were most often observed in long-term, population-scaled initiatives, particularly those strengthening physical activity opportunities and embedding actions across multiple settings (school, community, and health services). Parent-focused approaches may improve early feeding practices and parental self-efficacy but appear insufficient as standalone strategies, especially as children gain autonomy and environmental influences intensify. Technology-supported tools can enhance reach, personalization, and engagement, yet current evidence suggests they are most useful when integrated into broader prevention frameworks rather than implemented in isolation. Future European programs should prioritize early-life prevention, sustainability and implementation fidelity, equity-sensitive design (addressing socioeconomic and cultural barriers), and rigorous evaluation with standardized outcomes and longer follow-up to identify components that can deliver sustained and equitable reductions in childhood overweight and obesity.

## Figures and Tables

**Table 1:** Methodological characteristics and appraisal of included studies.

Program	Study Design Robustness	Control/Comparator	Sustainability of Effects	Key Methodological Concerns
Feel4Diabetes	High (cluster RCT)	RCT control	Partial; limited in obesity subgroup	Heterogeneity across countries
ToyBox	Moderate–High (cluster RCT)	RCT control	No sustained BMI effect	Behavioral change without adiposity effect
BigO	Moderate (prospective intervention study)	No control	Not assessed	No body composition outcomes
Med4Youth	Moderate (controlled clinical trial)	Non-randomized control	Short–medium term	Selected clinical sample
OCARIoT	Moderate (prospective intervention study)	No control	Reported reduction	Inconsistency between reported data and conclusions
PROBIT	Moderate (RCT)	RCT control	No significant anthropometric effect	Short follow-up
BOOST	Moderate–High (cluster RCT)	RCT control	Limited behavioral improvement; no long-term anthropometric data	Low parental exposure intensity; no BMI assessment
EPOC	High (RCT)	RCT control	No additional benefit beyond standard care	High baseline intervention intensity
Healthy School Start II	Moderate–High (cluster RCT)	RCT control	Not sustained at follow-up	Effects limited to subgroups
PRIMROSE	High (cluster RCT)	RCT control	No sustained effect	Long intervention duration with null anthropometric findings
Kids Obesity Prevention Game	Moderate (controlled school study)	Non-randomized control	Not sustained effect	Knowledge improved but no behavioral or BMI change
PETICA	Moderate (school-based controlled intervention)	Non-randomized control	Short-term behavioral effects	Anthropometric outcomes not primary endpoint
WAVES	High (cluster RCT)	RCT control	No effect	Null findings despite robust design
Slovenia PA	Moderate (population intervention study)	No control	Sustained over years	Non-randomized design
Gallotta et al.’s study [3]	Moderate (controlled school intervention)	Non-randomized control	Not assessed long-term	Small sample size; short duration
Drummy et al.’s, [4]	Moderate (controlled school study)	Non-randomized control	Short-term only	Very short intervention; no adiposity effect
AHEAD	Moderate (controlled school intervention)	Non-randomized control	No effect	Null findings despite adequate duration
Extra Fit!	Moderate–High (cluster RCT)	RCT control	No sustained effect	Null findings despite theory-based design and adequate duration
THAO	Moderate (population trend analysis)	No control	Long-term trend reduction	Observational design
VIF	Moderate (community longitudinal study)	No control	Long-term	No control group
Minority-focused program	Moderate (community-based controlled study)	Non-randomized control	Not clearly sustained	Small sample size; measurement limitations
National e-Health	Moderate (prospective evaluation study)	No control	1-year reduction	Resource-intensive
MINISTOP/MINISTOP 2.0	Moderate (RCT)	RCT control	Short-term behavioral effects only	No BMI effect

**Table 2:** Characteristics and effectiveness of childhood obesity prevention programs implemented in Europe (2015–2024).

Intervention Type	Country (Study Cohort)	Program (Author et al., Year)	Target Age Group	Main Setting	Baseline Sample Size (n)	Duration	Theoretical/Conceptual Basis	Main Outcomes on Behaviors	Effects on Anthropometric Outcomes	Key Limitations
Multinational/international	Bulgaria, Hungary, Belgium, Finland, Greece, Spain	Feel4Diabetes [5]	Children and families	Schol, community	n ≈ 11.396 families (baseline across countries)	2 years	Community-based lifestyle intervention; socio-ecological framework; diabetes-risk prevention model	↑ breakfast and fruit intake in some countries	↓ BMI z-score only in children with overweight; no effect in obesity	Limited effect on PA; heterogeneous results
Multinational/international	Belgium, Bulgaria, Germany, Greece, Poland, Spain	ToyBox [6]	Preschool (<6 y)	Kindergarten	n = 4.705 preschool children	6 months	Theory-driven behavioral intervention; kindergarten-based environmental modification model	↓ sugar-sweetened beverage intake	No consistent BMI effects	Substitution effects (↓ milk intake)
Multinational/international	Greece (Athens)	BigO [7]	Children and adolescents	School, home, healthcare	n = 427 children/adolescents	6 months	Personalized, data-driven lifestyle management; digital monitoring and feedback model	↑ water intake, ↓ snacking during screen time	Not primary outcome	Behavioral changes without body composition data
Multinational/international	Italy Med4Youth is a multinational European project (Portugal, Spain, Italy); however, the publication included in this review reports results from the Italian cohort only.	Med4Youth [8]	Adolescents with obesity	Clinical/dietary	n = 80 adolescents	4 months	Clinical dietary intervention; Mediterranean diet vs. low-fat comparative model	Improved diet quality	↓ BMI, ↓ body weight, ↓ BP (MD vs. low-fat)	Selected clinical population
Multinational/international	Spain, Greece and Brazil	OCARIoT [9]	School-aged children (mixed weight status)	School, home, healthcare	n = 127 children (Phase 2 final evaluation)	6 months	IoT-supported multidisciplinary obesity management; digital decision-support system	↑ lifestyle adherence	Reduction in obesity prevalence within the intervention sample	Technology-intensive, high resources
Parent-focused	Italy	PROBIT [10]	Infants–toddlers	Home/primary care	n = 300 mother–child dyads	24 months	Responsive feeding model; early-life behavioral prevention framework	↑ responsive feeding	No significant effect on overweight	Short follow-up
Parent-focused	Denmark	BOOST [11]	Adolescents	School/home	n = 1.013 adolescents	9 months	School-based multi-component intervention with parental involvement	↑ fruit and vegetable intake	Not assessed	Low parental exposure
Parent-focused	Germany	EPOC [12]	7–13 y with obesity	Clinical	n = 686 children	Inpatient program + 6 months follow-up (approx. 12 months total evaluation)	Behavioral parent-training integrated into clinical obesity treatment	↑ lifestyle, ↑ QoL	No added BMI benefit vs. control	High baseline intervention intensity
Parent-focused	Sweden	Healthy School Start II [13]	Preschool–early school	School/home	n = 378 children	6 months intervention (4-year follow-up reported)	Motivational and family-support model embedded in school setting	Minor subgroup effects	No sustained BMI effect	Effects not durable
Parent-focused	Sweden	PRIMROSE [14]	Preschool children	Primary child healthcare	n = 1.355 children	4 years	Motivational interviewing-based parental support model	No significant behavioral changes	No significant effect on BMI	No sustained effect despite long duration
School-based	Germany	Kids Obesity Prevention Game [15]	Primary school	School	n = 821 children	6 months	Serious game–based behavioral education model	↑ nutrition knowledge	No behavioral/BMI change	Knowledge–behavior gap
School-based	Croatia	PETICA [16]	Primary school	School	n = 1.106 children	5 months	School-based health education and physical activity promotion framework	↑ PA, ↑ breakfast and vegetables	Not primary outcome	Rural–urban differences
School-based	United Kingdom (West Midlands)	WAVES [17]	6–7 y	School	n = 1.467 children	12 months intervention (30-month follow-up)	Multi-component school lifestyle intervention model	No significant changes	No BMI effect	Insufficient intensity
School-based/population PA	Slovenia	Slovenia PA Program [18]	6–14 y	School/region	n = 34.387 children (population-level dataset)	3–4 years exposure	Population-scaled physical activity policy-driven intervention	↑ sustained PA	↓ BMI, ↓ obesity prevalence	Requires long-term policy support
School-based	Italy	[3]	Primary school children	School region	n = 106 children/adolescent	5 months	Combined physical education and nutrition education model	↑ PA; ↓ sedentary behavior	No significant improvement in body fat	Small sample; short duration
School-based	Northern Ireland (UK)	Classroom activity break study [4]	Primary school children	School	n = 120 children	12 weeks	Teacher-led structured classroom activity break model	↑ weekday MVPA	No BMI change; ↑ skinfold thickness	Very short duration; no adiposity improvement
School-based	England (UK)	AHEAD [19]	Adolescents (12–13 y)	School (peer-led)	n = 928 adolescents	12 months	Peer-led behavioral lifestyle intervention model	No significant improvements	No BMI effect	Ineffective peer-led structure; null results
School-based	Netherlands	Extra Fit! [20]	Primary school children (9–11 years)	School	n = 1.108 children	12 months	Theory-based nutrition and physical activity education program (self-regulation and behavioral change framework)	No significant long-term improvement in energy balance–related behaviors	No significant effect on BMI or overweight prevalence	Limited effectiveness despite theory-based design
Community-based	Spain (Madrid)	THAO Salud Infantil [21]	3–12 y	Community	n = 27.686 children	10-year trend evaluation (2009–2019)	Municipality-level community obesity prevention strategy	↑ healthy habits	↓ overweight and obesity trends	SES-related inequalities
Community-based	France	VIF Program [22]	Preschool–primary	Community	n = 8071 children	7 years	Long-term city-based environmental health promotion model	↑ lifestyle behaviors	↓ overweight and obesity	High implementation demands
Community-based	United Kingdom (London)	Minority-focused program [23]	Minority children	Schools and faith settings	n = 148 children	6 months	Community-based participatory research (CBPR) approach	Good acceptability	Not primary outcome	Measurement challenges
Healthcare-based	Greece	National e-Health Program [24]	2–18 y	Healthcare system	n = 2400 children and adolescents	1 year	Multidisciplinary healthcare-integrated obesity management system	↑ diet and PA adherence	↓ obesity (−32%), ↓ overweight	Resource-dependent
Technology-based	Sweden	MINISTOP/MINISTOP 2.0 [25,26]	Preschool	Home/healthcare	n = 315 children n = 552 children	6 months	mHealth behavioral intervention integrated into primary child healthcare	↑ composite lifestyle score	No BMI effect	Short duration

Abbreviations: BMI, body mass index; BMI z-score, body mass index z-score; BP, blood pressure; e-Health, electronic health; IoT, Internet of Things; MD, Mediterranean diet; MVPA, moderate-to-vigorous physical activity; PA, physical activity; QoL, quality of life; SES, socioeconomic status; y, years. Symbols: ↑ increase/improvement; ↓ decrease/reduction.

## Data Availability

No new data were created or analyzed in this study.

## Abbreviations

AHEAD	Activity and Healthy Eating in ADolescence
BMI	Body Mass Index
BMI-SDS	Body Mass Index Standard Deviation Score
BMI z-score	Body Mass Index z-score
BP	Blood Pressure
EPOC	Empowering Parents of Obese Children
e-Health	electronic health
HENRY	Health, Exercise, Nutrition for the Really Young
IoT	Internet of Things
mHealth	mobile health
MINISTOP	Mobile-based intervention intended to stop obesity in preschool-aged children (trial name)
MVPA	Moderate-to-Vigorous Physical Activity
OCARIoT	program name (acronym not expanded in the manuscript)
PA	Physical Activity
PETICA	PETICA—Play for Health (program name; acronym not expanded in the manuscript)
PRIMROSE	trial name (acronym not expanded in the manuscript)
PROBIT	Prevention of Obesity in Toddlers (trial name)
QoL	Quality of Life
RCT	Randomized Controlled Trial
SES	Socioeconomic Status
THAO	THAO Salud Infantil (program name; acronym not expanded in the manuscript)
VIF	VIF programme (program name; acronym not expanded in the manuscript)
WAVES	trial name (acronym not expanded in the manuscript)

## References

[1] Jebeile H., Kelly A.S., O’Malley G., Baur L.A. (2022). Obesity in children and adolescents: Epidemiology, causes, assessment, and management. Lancet Diabetes Endocrinol..

[2] Simmonds M., Llewellyn A., Owen C.G., Woolacott N. (2016). Predicting adult obesity from childhood obesity: A systematic review and meta-analysis. Obes. Rev..

[3] Gallotta M.C., Iazzoni S., Emerenziani G.P., Meucci M., Migliaccio S., Guidetti L., Baldari C. (2016). Effects of combined physical education and nutritional programs on schoolchildren’s healthy habits. PeerJ.

[4] Drummy C., Murtagh E.M., McKee D.P., Breslin G., Davison G.W., Murphy M.H. (2016). The effect of a classroom activity break on physical activity levels and adiposity in primary school children. J. Paediatr. Child Health.

[5] Manios Y., Androutsos O., Lambrinou C.-P., Cardon G., Lindstrom J., Annemans L., Mateo-Gallego R., de Sabata M.S., Iotova V., Kivela J. (2018). A school- and community-based intervention to promote healthy lifestyle and prevent type 2 diabetes in vulnerable families across Europe: Design and implementation of the Feel4Diabetes-study. Public Health Nutr..

[6] Pinket A.-S., Van Lippevelde W., De Bourdeaudhuij I., Deforche B., Cardon G., Androutsos O., Koletzko B., Moreno L.A., Socha P., Iotova V. (2016). Effect and process evaluation of a cluster randomized control trial on water intake and beverage consumption in preschoolers from six European countries: The ToyBox-study. PLoS ONE.

[7] Ioannou G., Petrou I., Manou M., Tragomalou A., Ramouzi E., Vourdoumpa A., Genitsaridi S.-M., Kyrkili A., Diou C., Papadopoulou M. (2024). Dietary and physical activity habits of children and adolescents before and after the implementation of a personalized intervention program for the management of obesity. Nutrients.

[8] Petraroli M., Shulhai A.M., Messina G., Rossi A., Bertolotti E., Esposito S.M., Scazzina F., Street M. (2023). First results from the ongoing Med4Youth European study: Comparing Mediterranean diet with a low-fat diet for adolescents with obesity. Horm. Res. Paediatr..

[9] Bastida L., Cea G., Moya A., Gallego A., Gaeta E., Sillaurren S., Barbosa P., Souto S., Rodrigues E., Torrego-Ellacuría M. (2023). Promoting obesity prevention and healthy habits in childhood: The OCARIoT experience. IEEE J. Transl. Eng. Health Med..

[10] Morandi A., Tommasi M., Soffiati F., Destro F., Grando F., Simonetti G., Bucolo C., Alberti E., Baraldi L., Chiriacò A. (2019). Prevention of obesity in toddlers (PROBIT): A randomised clinical trial of responsive feeding promotion from birth to 24 months. Int. J. Obes..

[11] Jørgensen S.E., Jørgensen T.S., Aarestrup A.K., Due P., Krølner R. (2016). Parental involvement and association with adolescents’ fruit and vegetable intake at follow-up: Process evaluation results from the multi-component school-based Boost intervention. Int. J. Behav. Nutr. Phys. Act..

[12] Warschburger P., Kroeller K., Haerting J., Unverzagt S., van Egmond-Fröhlich A. (2016). Empowering parents of obese children (EPOC): A randomized controlled trial on additional long-term weight effects of parent training. Appetite.

[13] Norman Å., Zeebari Z., Nyberg G., Elinder L.S. (2019). Parental support in promoting children’s health behaviours and preventing overweight and obesity: A long-term follow-up of the cluster-randomised Healthy School Start Study II trial. BMC Pediatr..

[14] Enö Persson J., Bohman B., Tynelius P., Rasmussen F., Ghaderi A. (2018). Prevention of childhood obesity in child health services: Follow-up of the PRIMROSE trial. Child. Obes..

[15] Mack I., Reiband N., Etges C., Eichhorn S., Schaeffeler N., Zurstiege G., Gawrilow C., Weimer K., Peeraully R., Teufel M. (2020). The Kids Obesity Prevention Program: Cluster randomized controlled trial to evaluate a serious game for the prevention and treatment of childhood obesity. J. Med. Internet Res..

[16] Cobal S., Bender D.V., Kljusurić J.G., Rumora Samarin I., Krznarić Ž. (2024). Effect of school-based educational intervention on childhood obesity in Croatian urban and rural settings. Children.

[17] Adab P., Pallan M.J., Lancashire E.R., Hemming K., Frew E., Barrett T., Bhopal R., Cade J.E., Canaway A., Clarke J. (2018). Effectiveness of a childhood obesity prevention programme delivered through schools, targeting 6- and 7-year-olds: Cluster randomised controlled trial (WAVES study). BMJ.

[18] Jurić P., Jurak G., Morrison S.A., Starc G., Sorić M. (2023). Effectiveness of a population-scaled, school-based physical activity intervention for the prevention of childhood obesity. Obesity.

[19] Bell S.L., Audrey S., Cooper A.R., Noble S., Campbell R. (2017). Lessons from a peer-led obesity prevention programme in English schools. Health Promot. Int..

[20] Kocken P.L., Scholten A.M., Westhoff E., De Kok B.P., Taal E.M., Goldbohm R.A. (2016). Effects of a theory-based education program to prevent overweightness in primary school children. Nutrients.

[21] Puga A.M., Carretero-Krug A., Montero-Bravo A.M., Varela-Moreiras G., Partearroyo T. (2020). Effectiveness of community-based intervention programs in childhood obesity prevention in a Spanish population according to different socioeconomic school settings. Nutrients.

[22] Vanhelst J., Deken V., Boulic G., Raffin S., Duhamel A., Romon M. (2021). Trends in prevalence of childhood overweight and obesity in a community-based programme: The VIF programme. Pediatr. Obes..

[23] Maynard M., Baker G., Harding S. (2017). Exploring childhood obesity prevention among diverse ethnic groups in schools and places of worship: Recruitment, acceptability and feasibility of data collection and intervention components. Prev. Med. Rep..

[24] Tragomalou A., Moschonis G., Kassari P., Papageorgiou I., Genitsaridi S.-M., Karampatsou S., Manios Y., Charmandari E. (2020). A national e-health program for the prevention and management of overweight and obesity in childhood and adolescence in Greece. Nutrients.

[25] Nyström C.D., Sandin S., Henriksson P., Henriksson H., Trolle-Lagerros Y., Larsson C., Maddison R., Ortega F.B., Pomeroy J., Ruiz J.R. (2017). Mobile-based intervention intended to stop obesity in preschool-aged children: The MINISTOP randomized controlled trial. Am. J. Clin. Nutr..

[26] Alexandrou C., Henriksson H., Henström M., Henriksson P., Delisle Nyström C., Bendtsen M., Löf M. (2023). Effectiveness of a smartphone app (MINISTOP 2.0) integrated in primary child health care to promote healthy diet and physical activity behaviors and prevent obesity in preschool-aged children: Randomized controlled trial. Int. J. Behav. Nutr. Phys. Act..

[27] Bryant M., Burton W., Collinson M., Martin A., Copsey B., Groves-Williams D., Foster A., Willis T.A., Garnett P., O'CAthain A. (2024). Effectiveness and cost-effectiveness of a sustainable obesity prevention programme for preschool children delivered at scale ‘HENRY’ (Health, Exercise, Nutrition for the Really Young): Protocol for the HENRY III cluster randomised controlled trial. BMJ Open.

[28] Patterson E., Nyberg G., Norman Å., Schäfer Elinder L. (2024). Universal Healthy School Start intervention reduced the body mass index of young children with obesity. Acta Paediatr..

